# Evaluation of Yield and Physicochemical Quality of *Pentadesma butyracea* Butter Obtained by Different Traditional Extraction Methods in Ghana

**DOI:** 10.1155/2024/5282230

**Published:** 2024-04-05

**Authors:** Josephine Akuba Timtey, Francis Alemawor, William Otoo Ellis, Jacob K. Agbenorhevi, Nana Baah Pepra-Ameyaw

**Affiliations:** Department of Food Science and Technology, Faculty of Biosciences, College of Science, Kwame Nkrumah University of Science and Technology, Kumasi, Ghana

## Abstract

*Pentadesma butyracea* seed butter or fat (PBSB) is a vegetable fat extracted from the seeds of the *P. butyracea* plant. The butter has potential use in the food, pharmaceutical, and cosmetic industries. The study investigated traditional PBSB processing methods in Ghana and evaluated their effects on yield, physicochemical parameters, and fatty acid composition. Four traditional extraction methods were identified and evaluated, and these methods are direct wet extraction of PBSB from a fresh mixture of the seed paste and water (DEW); wet extraction of PBSB from a 12-hour fermented mixture of the seed paste and water (FWO); direct wet extraction of PBSB from a fresh mixture of the seed paste, salt, and water (DES); and wet extraction of PBSB from a 12-hour fermented mixture of the seed paste, salt, and water (FSO). Results of physicochemical properties of the PBSB samples showed moisture content of 0.06-0.07%, free fatty acid of 1.38-2.43%, iodine value of 56.50-56.85 Wijs, peroxide value of 5.58-8.52 mEq/kg, relative density of 0.91, refractive index of 1.462-1.464, percent impurities of 0.015-0.017%, saponification value of 165.57-178.02 mg KOH/g, and percent unsaponifiable matter of 2.60-3.18%. The PBSB yield varied in the range of 21.68-26.97%, with the highest average butter yield observed for FWO. Seventeen fatty acids were characterized in the PBSB samples, and they included ten saturated fatty acids, five monounsaturated fatty acids, and two polyunsaturated fatty acids. Key fatty acids found in the PBSB samples were oleic acid (51.21-51.31%), stearic acid (43.22-43.33%), palmitic acid (2.91-3.07%), linoleic acid (0.49-0.51%), linolenic acid (0.12-0.20%), and arachidic acid (0.14-0.15%). PBSB samples produced by the various traditional extraction methods in Ghana recorded similar physicochemical characteristics as unrefined shea butter per the Regional Standard for Unrefined Shea Butter (CXS 325R-2017) as well as Cook Brand Margarine, a common commercial baking fat, and thus, their potential food application such as an alternative shortening/ingredient could be explored in a future study.

## 1. Introduction

*Pentadesma butyracea*, also called the butter tree, bears fat-rich seeds. The seed fat has medicinal, nutritional, sociocultural, economic, cosmetic, and pharmaceutical uses [1, 2]. The seeds have a fat content of about 50% with enhanced characteristics compared to shea butter regarding solidification point, slip point, saponification number, and fatty acid composition [3, 4]. Fat extraction can be done by chemical or physical methods. *P. butyracea* seed butter or fat (PBSB) extraction is carried out by women processors in rural communities and happens to generate substantial income for various households in Ghana and Benin [1]. Traditionally, woman processors employ the wet extraction method in PBSB extraction [5, 6]. The physical extraction of PBSB involves procedures such as seed drying, crushing and roasting, grinding, kneading, churning, boiling, filtration, and cooling [4, 5]. The crushing or cracking and milling enhance oil extraction while the heat treatment improves sensory characteristics and cell emulsion rupture and decreases oil viscosity. These increase oil fluidity, decrease oil surface tension, accelerate oil extraction, and improve sensory characteristics [7, 8]. The quality and consumer value of an oil/fat in terms of susceptibility to oxidation and production of undesirable volatile compounds can be influenced by the extraction method used (which can vary in terms of unit operations) and its chemical composition. In an earlier survey, [9] identified four traditional wet extraction methods for PBSB production, and these are direct wet extraction of PBSB from a fresh mixture of the seed paste and water (DEW); wet extraction of PBSB from a 12-hour fermented mixture of the seed paste and water (FWO); direct wet extraction of PBSB from a fresh mixture of the seed paste, salt, and water (DES); and wet extraction of PBSB from a 12-hour fermented mixture of the seed paste, salt, and water (FSO). However, there is a dearth of literature information on the effects of these traditional methods used in Ghana on PBSB. Knowledge of the influence of the extraction methods would be useful in optimizing the traditional processing of the butter with enhanced value in terms of yield, commercialization, physicochemical characteristics, and oxidative stability. Thus, the study sought to evaluate how the yield and quality characteristics of PBSB are affected by the various traditional extraction methods used in Ghana for its production.

## 2. Materials and Methods

### 2.1. Sampling Sources

**Figure 1:**
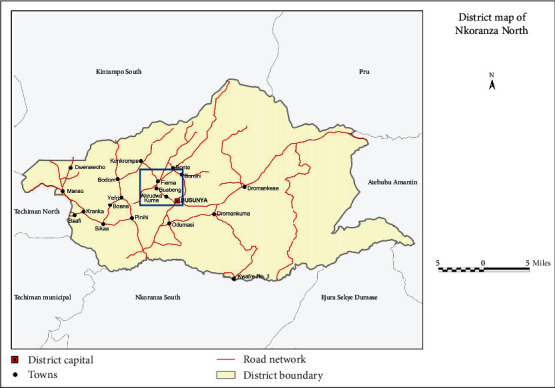
Map showing the study area (blue-boxed) in the Nkoranza North District in the Bono East Region of Ghana (source: Ghana Statistical Service, GIS; https://www2.statsghana.gov.gh/docfiles/2010_District_Report/Brong%20Ahafo/NKORANZA%20North.pdf).

*Pentadesma butyracea* fruits or berries (that had fallen under the trees) were picked in the forest of Akrudwa No. 1 (aka Akrudwa Panyin), Boabeng Fiema, Busunya, and Akrudwa No.2 (aka Akrudwa Kuma). These four (4) communities are located in the Nkoranza North Municipality of the Bono East Region of Ghana. These communities were selected because of their long-standing history of the use of *Pentadesma butyracea* [3].  Figure 1 shows the map of selected communities where the study took place.

### 2.2. Sample Preparation for P. butyracea Seed Butter or Fat (PBSB) Extraction

The *P. butyracea* seeds were depulped (removed from the dried berries) and sorted out, and all debris were removed. The initial moisture content was determined using standard [10] method 930.15. The seeds were sun-dried for 21 days to reduce the moisture content till a constant moisture content was obtained. After this, the seeds were washed with hot water at 100°C to get rid of all debris. They were then sun-dried for 6 hours and packed into jute bags for use in butter processing. Before butter processing, the seeds were cracked using a stone on a flat surface to reduce the sizes. The cracked seeds were dry-roasted on a traditional tripod for 40 minutes till they became dark and brittle. The roasted seeds were then milled using the locally designed attrition mill to obtain *P. butyracea* seed paste.

### 2.3. Traditional Methods Used for P. butyracea Seed Butter or Fat (PBSB) Extraction

The butter extractions were carried out with the guidance and support of some of the woman processors, ensuring that the sequence of unit operations for each extraction method was carefully and efficiently followed as done by the local processors. However, a few measures added included monitoring the duration of each unit operation and accurate measurement of weights/volumes of processing materials (water, salt, etc.), thus ensuring that the same durations and quantities were applied across replicate extractions for each traditional method; observing/practicing general hygiene and GMPs for all 4 methods; and sampling from the same homogenous seed paste stock for each traditional extraction method. Working together with the local processors and implementing the above-mentioned measures were part of efforts to obtain optimal yield of clean fat/butter and collect reliable samples/data. The procedures for the four (4) traditional wet extraction methods (DEW, DES, FWO, and FSO) identified by [9] are outlined in the following sections. These extraction methods vary in the pretreatment of the seed paste.

#### 2.3.1. Direct Extraction of *PBSB* from Seed Paste-Water Mixture (DEW)

**Figure 2:**
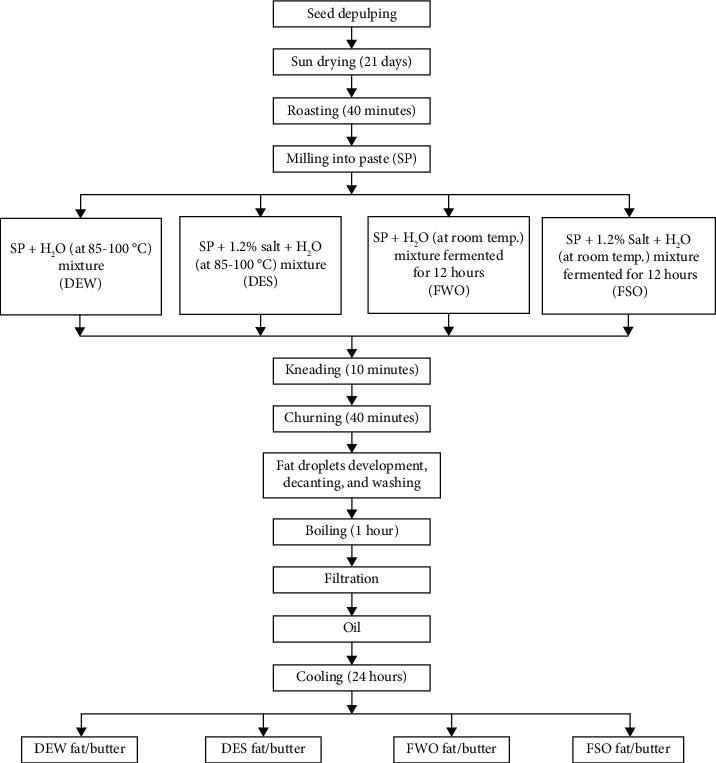
Flow diagram of the traditional methods for *P. butyracea* fat processing in Ghana.

**Figure 3:**
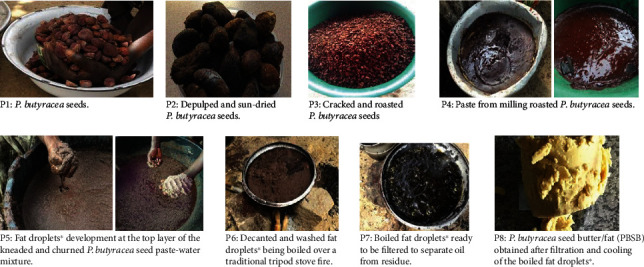
Photographs (P1 → P2 → P3 → P4 → P5 → P6 → P7 → P8) of some major steps in *P. butyracea* seed processing into fat/butter (PBSB). ^∗^Fat droplets consist of mainly *P. butyracea* seed fat and some nonoil *P. butyracea* seed residue.

A mixture of 14.5 kg of *P. butyracea* seed paste and 10 L of hot water (at 85-100°C) was prepared, and after 15-20 minutes of rest, when the temperature of the mixture was at *ca*. 40°C, the mixture was kneaded comfortably by hand for 10 minutes. The mixture was then churned vigorously by hand, with room temperature pipe-borne water (20 L) added to make the mixture fluffy. The water was added in small portions periodically while churning. After 40 minutes of churning, creamy fat droplets developed from the sides of the mixing bowl and covered the surface of the mixture. An extra 10 L of water (at room temperature) was added to the mixture to cause the fat droplets to clump together on the surface of the mixture. The fat droplets were then decanted and washed severally using 23 L of water (at room temperature) to remove excess residues. The washed fat droplets were then boiled, cooled, and filtered through 8 layers of white polyester material lining a funnel to obtain the *P. butyracea* fat, DEW (Figures 2 and 3).

#### 2.3.2. Direct Extraction of PBSB from Seed Paste-Water-Salt Mixture (DES)

A mixture of 14.5 kg of *P. butyracea* seed paste, 174 g of table salt (Annapurna® Iodated Salt, Unilever Ltd.), and 10 L of hot water (at 85-100°C) was prepared. The mixture obtained was further processed as described in Section 2.3.1, which included kneading and churning, as well as fat droplet collection/decanting, washing, boiling, and cooling, followed by filtration, to obtain the yellow *P. butyracea* fat, DES (Figures 2 and 3).

#### 2.3.3. Extraction of PBSB from a 12-Hour Fermented Seed Paste-Water Mixture (FWO)

A mixture of 14.5 kg of *P. butyracea* seed paste and 3 L of water (at room temperature) was prepared. The mixture was made to stand overnight for 12 hours (fermentation). The mixture was then mixed with 10 L of hot water and stirred. The mixture obtained was further processed as described in Section 2.3.1, which included kneading and churning (the volume of room temperature water used during this step was 17 L), as well as fat droplet collection, washing, boiling, and cooling, followed by filtration, to obtain the *P. butyracea* fat, FWO (Figures 2 and 3).

#### 2.3.4. Extraction of PBSB from a 12-Hour Fermented Seed Paste-Water-Salt Mixture (FSO)

A mixture of 14.5 kg of *P. butyracea* seed paste, 174 g of table salt (Annapurna® Iodated Salt, Unilever Ltd.), and 3 L of water (at room temperature) was prepared and made to stand overnight for 12 hours. The mixture was then mixed with 10 L of hot water and stirred. Similarly, the mixture obtained was further processed as described in Section 2.3.1, which included kneading and churning (the volume of room temperature water used during this step was 17 L), as well as fat droplet collection, washing, boiling, and filtration of the fat, to obtain the *P. butyracea* fat, FSO (Figures 2 and 3).

### 2.4. Analyses of Samples

The yield, physicochemical/compositional characteristics, and fatty acid profile of the PBSB samples obtained by the different extraction methods were determined. Each determination was replicated twice, and the mean value was recorded. The standard methods followed to determine the parameters have been briefly described as follows.

#### 2.4.1. Moisture Content Determination

Moisture content was determined by the oven drying method [11]. The sample of known weight was dried at 105°C ± 2°C for 3 hours, cooled, and weighed. The loss of mass was calculated as a percentage (% moisture content) using

(1) $$\begin{array}{c} \text{Moisture }\left(\%\right)=\frac{\left(\text{CW}+\text{WS}\right)-\left(\text{CW}+\text{DS}\right)}{\text{WS }}\times 100, \end{array}$$

where CW is the weight of moisture can (g), WS is the weight of sample taken (g), and DS is the weight of sample dried sample (g).

#### 2.4.2. Free Fatty Acid (FFA) Determination

Free fatty acid (FFA) was determined by a standard method [12]. The PBSB sample was dissolved by ethanol and diethyl ether, and the acids present were titrated with a known concentration of an alkaline solution. A blank test was performed along with the sample analysis. The FFA content was determined using Equations (2a) and (2b).

(2a) $$\begin{array}{c} \text{Acid value}\left(\text{mg KOH}/\text{g}\right)=\frac{\left(T-B\right)\times 5.61}{\text{weight of sample taken}}, \end{array}$$

where *T* is the titre volume of the sample (ml) and *B* is the titre volume of the blank (ml). The acidity or FFA was calculated as oleic acid (0.1 N NaOH ≡ 0.0282 g oleic acid), in which case,

(2b) $$\begin{array}{c} \text{FFA}=\text{acid value}\times 0.5. \end{array}$$

#### 2.4.3. Peroxide Value (PV) Determination

PV was determined by the iodometric method [13]. The PBSB sample portion, in solution in glacial acetic acid and chloroform, was treated with a solution of potassium iodide, and the liberated iodine was titrated with a standard volumetric sodium thiosulphate solution. A blank determination was carried out along with that of the test sample. The peroxide value was calculated using

(3) $$\begin{array}{c} \text{Peroxide value}\left(\text{meq}/\text{kg}\right)=\frac{\left(T-B\right)\times 0.01\times 1000}{\text{weight of sample taken}}, \end{array}$$

where *T* is the titre volume of the sample (ml) and *B* is the titre volume of the blank (ml). Duplicate determinations were carried out, and the mean value was recorded.

#### 2.4.4. Iodine Value Determination

The iodine value was determined using the Wijs method [14]. In this method, a test portion of the PBSB sample was dissolved in the solvent and the Wijs reagent was added. After a specified time, potassium iodide and water were added, and the liberated iodine was titrated with sodium thiosulphate solution. A blank determination (without the test sample) was carried out along with the test sample. The iodine value was calculated using

(4) $$\begin{array}{c} \text{Iodine value}\left(\text{Wijs}\right)=\frac{\left(B-A\right)\times 1.269}{\text{weight }\left(\text{in g}\right)\text{ of sample taken}}, \end{array}$$

where *A* is the titre volume of thiosulphate solution used (ml) and *B* is the titre volume for the blank (ml).

#### 2.4.5. Refractive Index Determination

The refractive index was determined using a refractometer (ATAGO, Model MASTER-PM 2313, Japan) according to a standard method [15]. The PBSB sample was melted and filtered using filter paper to remove impurities and trace moisture. A stream of water was circulated through the calibrated refractometer, and the temperature of the refractometer was adjusted to 44°C, and the prism was cleaned and dried. A few drops of the melted PBSB sample were placed on the prism closed and made to stand for 2 minutes. The refractometer and lighting were adjusted to obtain the most distinct reading possible, and the refractive index reading was taken.

#### 2.4.6. Relative Density Determination

The relative density of a fat/oil compares the weight of a given volume of the fat/oil to the weight of water of equal volume. The relative density of PBSB was determined according to the standard method [16] using a density bottle and an Ohaus analytical balance (model AS260D, Ohaus Corporation, USA). The relative density was calculated using

(5) $$\begin{array}{c} \text{Relative density}=\frac{\text{weight of sample }\left(\text{oil}\right)}{\text{weight of water}}. \end{array}$$

#### 2.4.7. Insoluble Impurity Determination

The insoluble impurity value of the PBSB sample was determined following a standard method [17]. A test portion of PBSB was treated with an excess of light petroleum ether (distillation range between 30°C and 60°C); then, the solution was filtered. The filter paper and residue were washed with the same solvent, then dried at 103°C ± 2°C, and weighed. The insoluble impurity percent was calculated using

(6) $$\begin{array}{c} \text{Insoluble impurities}=\frac{100\times \left(\text{wt}.\text{of filter paper}+\text{impurities}\right)-\left(\text{wt}.\text{of filter paper}\right)}{\text{wt}.\text{of sample taken}}. \end{array}$$

#### 2.4.8. Saponification Value Determination

The saponification value was determined according to a standard method [18]. The fat/oil sample (PBSB) was saponified by refluxing with a known excess of alcoholic potassium hydroxide solution. The alkali required for saponification was determined by titration of the excess potassium hydroxide with standard hydrochloric acid. The saponification value (S.V.) was calculated using

(7) $$\begin{array}{c} \text{S}.\text{V}.\left(\text{mg KOH}/\text{g}\right)=\frac{28.05\times \left(\text{blank titre value}-\text{sample titre value}\right)}{\text{weight of sample }\left(\text{g}\right)}. \end{array}$$

#### 2.4.9. Unsaponifiable Matter Determination

Unsaponifiable matter (USM) was determined by a standard method [19, 20]. The PBSB sample was saponified by boiling under reflux with an alcoholic potassium hydroxide solution. The unsaponifiable matter was extracted from the soap solution by diethyl ether. The solvent was evaporated, and the residue was weighed after drying and cooling. The percent unsaponifiable matter (% USM) was determined using

(8) $$\begin{array}{c} \text{USM }\left(\%\right)=\frac{\left(\text{wt}.\text{of flask with unsap}.–\text{wt}.\text{of empty flask}\right)}{\text{wt}.\text{of sample }\left(\text{g}\right)}\times 100. \end{array}$$

### 2.5. Yield of PBSB

The PBSB fat extracted from a known amount of dried *P. butyracea* seeds taken (i.e., 14.5 kg) was weighed. The percentage oil/fat yield was calculated using

(9) $$\begin{array}{c} \%\text{PBSB}/\text{fat yield}=\frac{\text{weight of fat extact}\left(\text{PBSB}\right)}{\text{weight of P}.\text{butyracea seeds taken}\left(\text{g}\right)}\times 100. \end{array}$$

### 2.6. Fatty Acid Methyl Ester Preparation and GC Analysis

Ten (10) milligrams of *Pentadesma butyracea* butter was dissolved in 1 mL toluene in a test tube. 200 *μ*L each of methyl nonadecanoate and butylated hydroxytoluene was added. An acidic methanol reagent of 2 mL was also added. The mixture was made to react overnight at 50°C in a stoppered test tube. The solution was allowed to cool, and 5 mL of sodium chloride was added. The fatty acid methyl esters (FAMEs) were extracted into 5 mL heptane twice. The combined heptane layers were washed with a 5 mL potassium bicarbonate solution. The heptane layer was dried over sodium sulphate.

The FAMEs in the heptane solution were analyzed using gas chromatography equipment coupled with a flame ionization detector (GC-FID). The GC oven equipped with BPX70-SGE capillary column (30 m length × 0.25 mm inner diameter × 0.25 *μ*m film thickness) was first set at 50°C and held for 2 min and then increased to 220°C at a ramp of 5°C/min and held for 4 min, to end up with a total runtime of 40 min. Helium was used as carrier gas at a flow rate of 1.5 mL/min (constant flow), and the injection volume was 1 *μ*L. The sample was transferred to the FID and was kept at 250°C with 300 mL/min zero air, 30 mL/min H_2_ flow, and 30 mL/min of makeup flow of N_2_. Star GC Workstation Version 6.4 chromatographic software (Varian) was used for data collection and calculation of all parameters (peak areas and retention times). The identification of FAs was based on a comparison of retention times with the FAMEs' standards.

### 2.7. Data Analysis

Data were presented as means ± standard deviation. Statistical Package for the Social Sciences (SPSS version 26.0) was used for the data analysis. The results were subjected to analysis of variance (ANOVA), and a comparison of treatment means was done by Tukey's test, adopting a significance level of 5% (*p* < 0.05).

## 3. Results and Discussion

### 3.1. Evaluation of Data Obtained

For each parameter determined, variations among the mean data recorded for the PBSB samples were evaluated to determine the effect of the traditional extraction methods on PBSB quality. Also, physicochemical characteristic mean data for the PBSB samples was compared with those of the Cook Brand® Margarine (CBM), as well as with the literature (range) values for unrefined shea butter [21].

CBM is a common commercial baking fat used in Ghana, and most consumers are used to baked products (commonly bread) made with CBM/margarine. Maximising or expanding the food value chain of PBSB is of paramount industrial interest. PBSB can potentially function as a baking fat or a fat stock/resource for shortening development. Thus, the comparison of PBSB samples extracted by the traditional methods to CBM, per the physicochemical data collected, was done to help determine which properties the PBSB samples share with CBM, and this is one of the preliminary steps to explore/guide PBSB potential (value-added) application and food security significance.

The sections that follow cover the details of the discussion/evaluation of the study data.

### 3.2. Evaluation of Physicochemical Indices of the Extracted PBSB Samples

#### 3.2.1. Moisture Contents of PBSB Samples

Table 1 shows the mean moisture contents (0.06-0.07%) for the PBSB samples obtained by the different traditional extraction methods. Moisture values for samples DEW, DES, FSO, and commercial CBM were similar (*p* > 0.05). However, the moisture content for the FWO butter was significantly lower (*p* < 0.05) than recorded for the other samples. Ayegnon et al. [5] reported an average moisture content of 0.65% for northern Benin *P. butyracea* butter extracted by traditional methods and 2.08% mean moisture for shea butter. PBSB shares similar characteristics as shea butter, and so based on the quality criteria by Africa Regional Standard and Code of Practice for Shea Kernels and Unrefined Shea Butter classification per moisture, the FWO butter falls within grade 1 (moisture max. limit of 0.05% for unrefined shea butter), while CBM, DES, DEW, and FSO samples can be categorized as grade 2 (moisture limit range of >0.05–0.2% for unrefined shea butter) [5, 21, 22]. Moreover, it was observed that the overnight (12 hours) fermentation treatments resulted in lower moisture content of the PBSB samples (FWO and FSO; Table 1). This could be due to the water molecules having enough time to interact strongly with polar groups in the nonoil components of the seed paste; hence, more residual water molecules are removed along with residue during the extraction steps [23]. High moisture contributes to the release or formation of FFAs leading to rancidity in the fats [24, 25]. Generally, the low moisture values recorded indicate that the PBSB samples would have a long storage life.

#### 3.2.2. Free Fatty Acid (FFA) Levels of PBSB Samples

The average FFA values for the *P. butyracea* butter samples analyzed were in the range of 1.37-2.43%, with the DEW butter recording the highest value of 2.43% and the lowest value of 1.38% observed for FWO butter (*p* < 0.05; Table 1). However, the FSO and DES butter samples were comparable to the commercial CBM, in terms of mean FFA (*p* > 0.05; Table 1). The author in [3] reported 4.2% FFA for *P. butyracea* fat/butter. Since *P. butyracea* butter is comparable to shea butter and so based on the quality criteria by Africa Regional Standard for Unrefined Shea Butter classification per %FFA, all the samples may be classified as grade 2 butter category (%FFA limit range of 1.11–3.00 for unrefined shea butter) and the butter in this category can be used in food industry applications [21, 22]. FFAs are derived from triacylglycerols by cleavage of ester bonds due to the hydrolytic action of lipase, high temperature, and/or moisture [26]. FFAs can act as prooxidants in oil systems by speeding up the rate of hydroperoxide decomposition. Thus, high FFA content in the oil may cause further oxidation, leading to the development of an objectionable taste and flavour in the oil. Percent FFA is often used to indicate the oil quality and its suitability for edible applications [26].

Differences observed among FFA values for the PBSB samples may have been influenced by the variations in the extraction/processing methods used. The application of salt in the fat extraction could have reduced available water molecules (low water activity) for lipolysis in the PBSB samples, as sodium and chloride ions from the salt (NaCl) can hold onto/associate with water molecules. This possibly contributed to the relatively low %FFA observed for DES and FSO as compared to values for DEW butter, although the lowest was observed in FWO butter. The low FFA values recorded indicate that the PBSB samples potentially will have good oxidative stability and will be useful in food applications [27]. FWO and DEW will, respectfully, exhibit the least and the highest susceptibility to rancidity development.

#### 3.2.3. Percent Insoluble Impurities of PBSB Samples

PBSB samples recorded mean percent insoluble impurities which varied in the range of 0.015-0.017%, and these are less than 0.021% recorded for the commercial CBM (Table 1). The DEW butter recorded the least average percent insoluble impurity level of 0.015%, which was significantly lower (*p* < 0.05) than that observed for the CBM. Per quality criteria used for unrefined shea butter, all the PBSB samples analyzed fall in the grade 1 category (0-0.09%, acceptable %insoluble impurities limit for unrefined shea butter), making them suitable for direct consumption, cosmetic, and pharmaceutical needs [21, 22]. Insoluble impurities in crude fat may result from the physical contact of the fat with packaging material, soil, or dust particles. It is an important quality parameter that could influence the deterioration of butter; for instance, metals especially iron can catalyse the oxidation of fat and reduce its market value [28]. Impurities can also contribute to an increase in the FFA content through the hydrolysis of ester linkages [24]. The low levels of impurities observed in the present study could also indicate the efficiency of the extraction methods for the PBSB samples.

#### 3.2.4. Peroxide Values (PVs) of PBSB Samples

Peroxide value (PV) ranged from the lowest of 5.52 mEq/kg for the FSO butter to the highest of 8.52 mEq/kg obtained for the DES butter (*p* < 0.05; Table 1). PV is an initial indication of rancidity or an indicator of oxidative spoilage in fats and oils. The higher the PV, the more susceptible the fat is to deterioration. The results obtained indicate that the application of salt (NaCl) increased lipid oxidation in the PBSB samples. The ability of salt as a prooxidant has been observed by other researchers, even though its mechanism is not fully explained [29, 30]. The 12-hour fermentation of the seed paste with salt resulted in the FSO butter recording the least PV compared to its counterpart, FWO, obtained by the 12-hour fermentation of the unsalted seed paste. Tunieva et al. [31] recorded a 17% reduction in PV when 2.0% table salt was added to back fat, compared with unsalted back fat. Also, antioxidant substances, including phenolics, that are inherent in the samples or those formed during fermentation in the presence of salt could have contributed to the reduced PV [32]. Lower PVs of 0.74 meqO2/kg and 2.09 meqO_2_/kg have been reported for *P. butyracea* butter and shea butter, respectively [5]. In another study, [33] reported 2.17 meqO2/kg and 3.78 meqO2/kg for *P. butyracea* butter and shea butter, respectively. Tchobo et al. [2] noted a higher PV of 17.30 meqO2/kg for *P. butyracea* butter. PVs from 0.84 to 7.32 meqO2/kg have been observed for *P. butyracea* butter samples extracted from boiled and dried kernels and 2.72 to 15.20 meqO2/kg in *P. butyracea* butter from seeds dried without boiling after 12 months of storage [6]. Differences in the PVs for the PBSB samples may have been influenced by the variations in processing/extraction methods used. Since all the PVs observed in the present study were in the range of 8.52-5.58 meqO_2_/kg, all the samples fall in the grade 1 category (10 meqO_2_/kg maximum PV for unrefined shea butter) per quality criteria for unrefined shea butter [21, 22]. This indicates that the PBSB samples can be directly consumed or applied in food product development.

#### 3.2.5. Iodine Values (IVs) of PBSB Samples

Table 1 indicates the iodine values (IVs) observed for the various PBSB samples, and they vary insignificantly in the range of 56.47-56.85 I_2_ g/100 g (*p* > 0.05). No significant differences were observed between the IVs for the various PBSB samples and those recorded for the commercial CBM. Previous studies have reported lower IVs of 39.15 I_2_ g/100 g and 42.06 I_2_ g/100 g for *P. butyracea* butter and shea butter, respectively [33]. For unrefined shea butter, the permissible limit ranges from 30 to 75 I_2_ g/100 g [21]. The iodine value (IV) determines the degree of unsaturation and the number of carbon-carbon double bonds in the fat indicating the extent of flow as well as the susceptibility of the oil toward oxidative rancidity or stability [34]. The IVs recorded for the PBSB samples in the present study are less than 100 I_2_ g/100 g. Oils with IV less than 100 I_2_ g/100 g of oil are nondrying oils, and the lower IV indicates a lower number of unsaturated bonds present; thus, such oils will have lower susceptibility to oxidative rancidity [35]. The results show that the IV for PBSB varied marginally irrespective of the traditional extraction method used.

#### 3.2.6. Relative Density Values of PBSB Samples

From Table 1, no significant differences (*p* > 0.05) were observed in the mean relative density of fat samples. The PBSB samples obtained by the various traditional methods of extraction as well as the commercial baking fat, CBM, recorded the same mean relative density value of 0.91 g/mL. However, mean density values for the various PBSB samples in the present study are lower than the density values (0.93-1.00 g/mL) reported for shea butter by [36], and the differences could be due to variations in composition and plant species for butter source. Relative density gives information about the unique identity of the butter, aiding in the identification of adulteration [37]. The relative densities obtained in this study are within the range of 0.89-0.93 g/mL recommended for a similar fat, unrefined shea butter [21].

#### 3.2.7. Refractive Indices (RIs) of PBSB Samples

The mean RIs for the samples varied in the range of 1.462-1.464, with the CBM and FSO samples recording the highest values while the DEW butter recorded the lowest value, which was closely followed by 1.463 for the FWO butter. Except for the DEW butter, the indices of the *P. butyracea* butter samples were comparable (*p* > 0.05) to those recorded for the commercial CBM (Table 1). The RIs observed in the current study are consistent with the normal range (1.462-1.465) set for classifying unrefined shea butter [21]. The observed RIs mean values are similar to those reported in previous studies on tree-nut butter, including Tchobo et al. [2] and Ayegnon et al. [5] who recorded a range of 1.460-1.462 for *Pentadesma butyracea* fat; while Garti et al. [38] reported 1.46 for shea butter. The refractive index (RI) is a physical property used in oil and fat identification and for checking the purity of fats and oils where there is suspicion of adulteration [39]. A high RI indicates a high chance of spoilage of the oil/fat due to oxidation. The RI is a basic value influenced by molecular weight, fatty acid, chain length, the degree of unsaturation, and the degree of conjugation, of the oil/fat [40].

#### 3.2.8. Unsaponifiable Matter (USM) Levels of PBSB Samples

Average USM values for the samples were in the range of 2.60-3.18%. It was also observed that the DES and DEW butter samples, which were produced by direct extraction of the traditional method, had a higher USM content (*p* < 0.05) compared to those of FWO and FSO butter samples that were extracted after 12-hour fermentation of the *P. butyracea* paste (Table 1). Moreover, the values for FWO and FSO samples were similar to those of the commercial CBM. The USM values we recorded for PBSB samples are higher than those (1.55% to 1.8%) reported by [2, 33] and [3]. Shea butter is reported to have a higher USM, between 4 and 11% [28]. The standard USM range for shea butter is 1-19% [21]. Lower USM levels have been reported for important solid fats in the food and cosmetic sectors such as palm kernel (≤10%), cocoa (0.5-1%), and coconut (0.2-0.4%) oils [41, 42]. USM includes those substances frequently found dissolved in fats and oils that cannot be saponified by the usual caustic treatment but are soluble in ordinary fat and oil solvents. Included in this group of compounds are higher aliphatic alcohols, sterols, pigments, and hydrocarbons [43]. Edible fat and oils usually have a small amount of USM, so a high value may indicate adulteration [44, 45]. Also, the higher the saponification value, the lower would be the USM value [46]. Variations in the processing methods used largely account for the differences in the values observed in the present study. The fermentation of the seed paste may have modified the properties of some unsaponifiable constituents enhancing their solubility in the extraction water medium, and this may have subsequently reduced the USM of the butter; i.e., during the fermentation, enzymatic activities could have resulted in the breakdown of some unsaponifiable compounds. Unsaponifiables may occur naturally or may be formed during the processing or degradation of the fat [47]. Fats with higher USM are preferred for cosmetic and medicinal purposes due to the availability of desirable secondary plant metabolites such as vitamins, as well as antioxidant and anti-inflammatory properties [28, 38]. The PBSB samples can be used for medicinal and cosmetic purposes.

#### 3.2.9. Saponification Values (SVs) of PBSB Samples

The mean SVs for the PBSB samples were in the range of 165.57-179.28 mg KOH/g, with DEW, DES, and FSO recording SVs similar to that for commercial CBM (*p* > 0.05), while FWO gave the least value (*p* < 0.05). Our recorded SVs are in line with SV range of 160.4-192.2 mg KOH/g reported for a similar fat, shea butter [38]; and also they fall within the standard SV range of 160-190 mg KOH/g for crude shea butter [21]. Higher SVs of 192.15 mg KOH/g and 187.99 mg KOH/g have been reported for *P. butyracea* butter and shea butter, respectively [33]. Saponification depicts the soap-making ability of fats. SV portrays the molecular weight or size as a function of the chain length of the constituent fatty acids; SV around 195 shows that oil contains mainly fatty acids of high molecular mass while the oils having high SVs (around 300) have mainly fatty acids of low molecular mass useful for soap making [48, 49]. The recorded SVs suggest that PBSB samples contained fatty acids of high molecular mass mainly suitable for consumption rather than soap making.

### 3.3. Yield of PBSB Extracted by Traditional Methods

The highest yield (26.97%) was recorded for the FWO butter sample, while the lowest yield (21.68%) was obtained for the FSO butter sample. Significant differences (*p* < 0.05) were observed among the yields of butter samples except between DES and DEW samples. Aïssi et al. [6] reported an oil yield of 25% for roasted *P. butyracea* seeds and 33.5% for the seeds boiled and dried before extraction. Also, yields of 9.73-31.20% have been reported for *P. butyracea* butter using traditional methods of extraction in northern Benin [50]. Adomako [3] reported a 50% fat yield for *P. butyracea* butter, while [2] recorded 41.9% fat content for *P. butyracea* seed. Also, Aïssi et al. [51] reported that fat content of traditionally processed *P. butyracea* butter has been reported in the range of 48.21-48.86%. Differences in PBSB yields could be attributed to variations in geographical location, genetic influence, and environmental factors to which the *P. butyracea* plant was exposed, as well as the type or efficiency of the fat extraction method employed. It is reported that early fruiting during the dry season, coupled with a high elevation and cool temperatures, is associated with high levels of fat production in shea kernels [52]. The sampling sites for the present study are settlements/rural communities located in the Nkoranza North District in the Bono East Region of Ghana. This district lies within longitude 10 10′ and 10 55′ west and latitude 70 20′ and 70 55′ north and is generally low lying and rises gradually from 153 m to 305 m above sea level. Nkoranza North District is found within the wet semiequatorial region and has mean annual rainfall range of 800–1200 mm and temperature high average annual temperature of about 26°C [53, 54].

### 3.4. Fatty Acid Profiles of PBSB Samples Extracted by Different Traditional Methods

Table 2 presents the fatty acid compositions for the various PBSB samples extracted by 4 traditional methods. A total of 17 fatty acids were identified, including ten saturated fatty acids (SFAs), five monounsaturated fatty acids (MUFAs), and two polyunsaturated fatty acids (PUFAs).

Previous works identified lower numbers of fatty acids in *P. butyracea* butter: [5] identified nine fatty acids, and [55] found seven fatty acids, while [3] reported six fatty acids. From Table 2, the PBSB samples had 47% SFAs and 52% unsaturated fatty acids (UFAs). The predominant SFA and UFA in the samples were oleic acid (51.22-51.34%) and stearic acid (43.23-43.33%), respectively, which together constitute 95% of the PBSB. These observations are similar to the findings by [56] who reported that *Pentadesma butyracea* fat was made up of 52.81% UFAs and 47.19% SFAs. Previous findings also show that stearic acid and oleic acid accounted for about 96% of the butter [2, 5]. The oleic acid values (above 51%) recorded for the PBSB samples are higher compared to 40-50% reported in palm oil [57] and 22.6% in soybean oil [58]. The high level of oleic acid, a monounsaturated fatty acid (MUFA), enhances the value of PBSB. MUFAs are vital in lowering the risk of coronary heart diseases as they aid in reducing low-density lipoprotein (LDL) cholesterol concentration in the blood. Mishra et al. [59] also found that oleic acids can reduce oxidative stress-induced disorders and offer protection against adrenaline-induced gastric injury, probably through their antioxidant mechanism(s).

Moreover, the fatty acid composition of PBSB indicates its potential food applications. The high percent value of stearic acid in PBSB gives its solid nature at room temperature, and so it can be used as a solid base stock in healthier margarine and shortening production, thus avoiding the *trans*-fat that comes with the use of partially hydrogenated hard stocks [60, 61]. PBSB can also serve as a good source of stearic acid, which can be used to produce stearates as well as function as emulsifiers and coating agents due to its hydrophilicity and lipophilicity properties, and these have applications in the food, cosmetic, and pharmaceutical industries. In humans, stearic acid is as potent as oleic acid at reducing plasma low-density lipoprotein (LDL) cholesterol levels, thus reducing cardiovascular risk [62].

Majority of the fatty acids identified in the PBSB samples were in very low quantities, ranging from 0.01% to 3.07% (Table 2), and they include SFAs, medium-chain fatty acids (MCFAs), and UFAs. The concentrations of MCFAs were either significantly lower or undetectable in the DES and DEW samples compared to their counterparts, FSO and FWO butter samples. Our recorded mean levels of these fatty acids were similar to previous findings obtained for *P. butyracea* butter and shea butter [2, 5, 38]. Among the UFAs identified were linoleic acid and linolenic acid, which are dietary essential fatty acids important for the production of leukotrienes (bronchoconstrictors), prostaglandins (potent vasodilators), thromboxanes (potent vasoconstrictors), and prostacyclins (potent vasodilators), and these biomolecules offer modest protection against cardiovascular diseases as well as control of inflammations [63, 64]. Higher levels of palmitic acid, myristic acid, and lauric acid have been associated with raised LDL cholesterol [65], and so the low levels we recorded for these acids potentially enhance the dietary value of the PBSB samples.

The odd-chain fatty acids (OCFAs), margaric acid (C17:0), and margaroleic acid (C17:1, *cis*-10) were identified in very low concentrations in the PBSB samples. Although these OCFAs are primarily of ruminant origin, literature also indicates that they are widely spread in nature and found in common foods and dietary fats including marine and freshwater fish, some vegetables, seaweeds, and several vegetable oils [66, 67]. Yang et al. [68] reported on the presence of odd-chain fatty acids in higher animals, plants, and microheterotrophs. In plants, these OCFAs are present in small quantities or as minor components (<1%). OCFAs, including margaric acid and margaroleic acid, also have been identified/reported in plants or tree nuts including walnuts [69, 70], hazelnuts [71, 72], almonds [73, 74], pecan nuts [74], olive oil [75], seed oils of *Thespesia populnea* or Portia tree and Gossypium hirsutum [76], kenaf seed oil [77], and red cabbage [78]. Moreover, [5] found 0.15-0.16% of heptadecanoic acid in *P. butyracea* butter, while [79] reported heptadecanoic acid of 0.07% in shea butter. While most scientific publications on fatty acid composition barely report on fatty acids in low or negligible quantities [66], it is significant to state that OCFAs including margaric acid (C17:0) and margaroleic acid (C17:1, *cis*-10) have been found to reduce inflammation, improve insulin sensitivity, and promote weight loss [80, 81]. Generally, the differences observed in the fatty acid compositions for the various PBSB samples are largely due to the variations in the traditional methods used for butter extraction/processing.

## 4. Conclusion

The study considered four traditional processing methods used for producing *P. butyracea* seed butter/fat (PBSB) in Ghana, and the objective was to evaluate their effects on its physicochemical quality and yield. Extraction of PBSB from the fermented mixture of the seed paste and water (FWO) produced the highest yield (26.97%) while the least yield (21.68%) was observed for the PBSB extracted from the fermented mixture of seed paste, salt, and water (FSO). Overall and comparatively, the PBSB obtained by the FWO method also showed better physicochemical quality in terms of the levels of FFA, PV, impurities, and fatty acid profile. Moreover, the various PBSB samples showed similar characteristics with shea butter and commercial baking fat, CBM, including levels of oleic acid and stearic acid. The findings from the present study add to existing scientific information on the extraction and characteristics of PBSB. Future studies would consider the optimization of the traditional methods, the molecular organization of PBSB fat crystals, and the shelf life of PBSB.

## Figures and Tables

**Table 1:** Physicochemical characteristics of four *P. butyracea* butter samples extracted by different traditional methods used in Ghana and a commercial baking fat, Cook Brand® Margarine.

Parameter	Fat/butter sample				
	^1^CBM	^2^DES	^3^FWO	^4^DEW	^5^FSO
Moisture (%)	0.08 ± 0.00^b^	0.07 ± 0.00^b^	0.06 ± 0.00^a^	0.07 ± 0.00^b^	0.07 ± 0.00^b^
FFA (%)	1.91 ± 0.01^b^	1.89 ± 0.01^b^	1.38 ± 0.01^a^	2.43 ± 0.02^c^	1.91 ± 0.02^b^
Iodine value (Wijs)	56.47 ± 0.05^a^	56.64 ± 0.00^a^	56.85 ± 0.11^a^	56.78 ± 0.01^a^	56.50 ± 0.01^a^
Peroxide value (mEq/kg)	6.61 ± 0.04^b^	8.52 ± 0.10^c^	6.62 ± 0.03^b^	6.61 ± 0.04^b^	5.58 ± 0.03^a^
Relative density	0.91 ± 0.00^a^	0.91 ± 0.00^a^	0.91 ± 0.00^a^	0.91 ± 0.00^a^	0.91 ± 0.00^a^
Refractive index (at 44°C)	1.464 ± 0.00^ab^	1.464 ± 0.00^ab^	1.463 ± 0.00^ab^	1.462 ± 0.00^a^	1.464 ± 0.00^b^
Impurities (%)	0.021 ± 0.00^b^	0.017 ± 0.00^ab^	0.016 ± 0.00^ab^	0.015 ± 0.00^a^	0.017 ± 0.00^ab^
Saponification value (mg KOH/g)	179.28 ± 0.05^b^	177.07 ± 0.58^b^	165.57 ± 0.07^a^	178.02 ± 0.52^b^	177.55 ± 1.05^b^
Unsaponifiable matter (%)	2.60 ± 0.04^a^	3.16 ± 0.01^b^	2.65 ± 0.02^a^	3.18 ± 0.10^b^	2.60 ± 0.04^a^
Yield (%)	—	25.53 ± 0.01^b^	26.97 ± 0.02^c^	25.53 ± 0.01^b^	21.68 ± 0.39^a^

Data are represented as mean ± standard deviation. Means with the same letters along rows are not significantly different (*p* > 0.05). ^1^CBM = Cook Brand® Margarine (common commercial baking fat)—a fully refined, partially hydrogenated oil/fat, containing other components including water, salt emulsifiers, mono- and diglycerides of fatty acids, soy lecithin, beta carotene, antioxidants (propyl gallate, BHA), vitamin A 26000 IU/kg, and vitamin D 3900 IU/kg. ^2^DEW = *P. butyracea* butter extracted just after mixing seed paste with water only. ^3^FWO = *P. butyracea* butter extracted after a 12-hour fermentation of seed paste mixed with water only. ^4^DES = *P. butyracea* butter extracted just after mixing seed paste with both water and salt. ^5^FSO = *P. butyracea* butter extracted after a 12-hour fermentation of seed paste mixed with both water and salt.

**Table 2:** Fatty acid composition (%) of four *P. butyracea* butter samples obtained by different traditional extraction methods.

Fatty acid	Fat/butter sample			
	DES	DEW	FSO	FWO
Caprylic acid (C8:0)	0.02 ± 0.00^a^	0.02 ± 0.00^a^	0.03 ± 0.00^b^	0.03 ± 0.00^b^
Capric acid (C10:0)	ND	ND	0.01 ± 0.00^a^	0.01 ± 0.00^a^
Lauric acid (C12:0)	ND	ND	0.09 ± 0.00^a^	0.09 ± 0.00^a^
Myristic acid (C14:0)	0.01 ± 0.00^a^	0.01 ± 0.00^a^	0.02 ± 0.00^b^	0.02 ± 0.00^b^
Palmitic acid (C16:0)	2.91 ± 0.00^a^	2.92 ± 0.00^b^	3.07 ± 0.00^c^	3.05 ± 0.00^d^
Palmitoleic acid (C16:1)	0.09 ± 0.00^a^	0.09 ± 0.00^a^	0.08 ± 0.00^b^	0.08 ± 0.00^b^
Margaric acid (C17:0)	0.14 ± 0.00^a^	0.14 ± 0.00^a^	0.14 ± 0.00^a^	0.14 ± 0.00^a^
Margaroleic acid (C17:1)	0.02 ± 0.00^a^	0.02 ± 0.00^a^	0.02 ± 0.00^a^	0.02 ± 0.00^a^
Stearic acid (C18:0)	43.23 ± 0.00^a^	43.31 ± 0.02^b^	43.33 ± 0.00^b^	43.33 ± 0.00^b^
Elaidic acid (C18:1n9t)	0.15 ± 0.00^a^	0.11 ± 0.06^a^	0.06 ± 0.00^a^	0.06 ± 0.00^a^
Oleic acid (C18:1n9c)	51.31 ± 0.00^a^	51.34 ± 0.08^a^	51.22 ± 0.00^a^	51.22 ± 0.01^a^
Linoleic acid (C18:2n6c)	0.50 ± 0.00^b^	0.51 ± 0.00^a^	0.50 ± 0.00^b^	0.50 ± 0.00^b^
Linolenic acid (C18:3n3)	0.13 ± 0.00^a^	0.13 ± 0.00^b^	0.20 ± 0.00^c^	0.12 ± 0.00^d^
Arachidic acid (C20:0)	0.15 ± 0.00^b^	0.14 ± 0.00^a^	0.14 ± 0.00^a^	0.14 ± 0.00^a^
Gondoic acid (C20:1)	0.04 ± 0.00^a^	0.04 ± 0.00^a^	0.05 ± 0.00^b^	0.05 ± 0.00^b^
Behenic acid (C22:0)	0.01 ± 0.00^a^	0.01 ± 0.00^a^	0.01 ± 0.00^a^	0.01 ± 0.00^a^
Lignoceric acid (C24:0)	0.01 ± 0.00^a^	0.01 ± 0.00^a^	0.01 ± 0.00^a^	0.06 ± 0.06^a^
Sums and ratio				
*Σ* SFAs	46.48	46.57	46.85	46.88
*Σ* UFAs	52.24	52.24	52.13	52.05
*Σ* MUFAs	51.61	51.60	51.43	51.43
*Σ* PUFAs	0.63	0.64	0.70	0.62
UFA/SFA	1.12	1.12	1.11	1.11

ND = not detected. Data are represented as mean ± SD. ^a-d^Means in the same row with different superscript letters were significantly different (*p* < 0.05). *Σ* SFAs = sum of saturated fatty acids; *Σ* UFAs = sum of unsaturated fatty acids; *Σ* MUFAs = sum of monounsaturated fatty acids; *Σ* PUFAs = sum of polyunsaturated fatty acids; UFAs/SFAs = ratio between unsaturated and saturated fatty acids.

## Data Availability

All data is provided in full in the results section of this paper.
